# Transitioning toward Sustainable Development Goals: The Role of Household Environment in Influencing Child Health in Sub-Saharan Africa and South Asia Using Recent Demographic Health Surveys

**DOI:** 10.3389/fpubh.2016.00087

**Published:** 2016-05-04

**Authors:** Ankit Anand, Nobhojit Roy

**Affiliations:** ^1^Population Research Centre, Institute for Social and Economic Change, Bangalore, India; ^2^Environmental Health Resource Hub, Tata Institute of Social Sciences, School of Habitat Studies, Mumbai, India

**Keywords:** household environment, child health, Sub-Saharan Africa, South Asia, MDG, DHS, SDG-6

## Abstract

The Millennium Development Goals are now replaced by 17 sustainable development goals. The emphasis of old goals was on improving water, sanitation, and child mortality conditions in developing countries. The study explored the major question about the association between different household environment conditions with child survival and health in Sub-Saharan African and South Asian countries in the current scenario. This paper estimated the risk of death, morbidity, and undernutrition among children living in households with the improved sources of water, sanitation, and non-solid cooking fuel. Two sources of information are explored in this study. First, data from World Health Statistics (WHS)-2014 for all of the Sub-Saharan African and South Asian countries were used. Second, available standard Demographic and Health Survey (DHS) performed in the countries of Sub-Saharan Africa and South Asia after 2010 was included in the study. It resulted in the inclusion of 15 countries which were Bangladesh (2011), Congo Republic (2013–2014), Cote d’Ivoire (2011–2012), Ethiopia (2011), Gambia (2013), Mali (2012–2013), Mozambique (2011), Namibia (2013), Nepal (2011), Niger (2012), Nigeria (2013), Pakistan (2012–2013), Sierra Leone (2013), Uganda (2011), and Zambia (2013). The scatter plot diagram was plotted, and the curve was fitted using the WHS-2014. Cox regression and logistic regression were used to estimate adjusted risks (odds ratio) of child mortality and health outcomes using DHSs. The use of non-solid cooking fuel was very high in most of the Sub-Saharan African and South Asian countries. There was a positive correlation between improving access to safe drinking water and sanitation. The exponential curve fitted well with child mortality and household environmental indicators. The use of improved source of water and sanitation significantly related with the lower odds ratio of death, morbidity, and undernutrition among children aged 12–59 months. The risks were not significant for children aged less than 12 months. The study provides evidence that these environmental conditions hold importance for improving child health, especially in Sub-Saharan African countries.

## Introduction

The UN Millennium Declaration was signed by 189 countries at the start of the twenty-first century and resulted into eight Millennium Development Goals (MDGs) (1). All these goals were related to each other and established to enhance the development effort in the developing countries. The MDGs are now replaced by 17 sustainable development goals (SDGs) (2). The emphasis of the MDGs was on improving water, sanitation, and decreasing child mortality, especially in Sub-Saharan African and South Asian countries. The new SDG-6 moved on from MDGs and included various aspects of water and sanitation (2, 3). The study assessed the association of the household environment with child health, important in the public health context of Sub-Saharan Africa and the South Asian countries.

Millennium Development Goal-4 emphasized on reducing infant mortality and under-five mortality rates in developing countries. It targeted to reduce it by two-thirds between 1990 and 2015 (1, 4). Since 1990, South Asian and Sub-Saharan regions had seen a significant decline in their child and infant mortality (5). The child mortality remains a grave concern in countries of South Asia and Sub-Saharan Africa (5, 6). According to the UNICEF 2011 report, globally 98.6% of deaths among children less than 5 years of age took place in the developing countries (5). Sub-Saharan Africa and South Asian countries contribute to more than 80% of the total child deaths in the world (6, 7). Acute respiratory tract infections remain the leading cause of deaths among children, followed by diarrheal diseases (8). Infectious diseases are widespread among children under 5 years in developing countries and can affect the development through direct and indirect pathways. Unhygienic sanitation practices and unsafe drinking water are some of its main causes (9–11). Underweight children are also at higher risk of diarrhea, malaria, and other childhood morbidities (12).

The environment surrounding the children can have a major effect on their survival and health (10, 11, 13). The World Health Organization (WHO) has defined environmental health as “all the physical, chemical, and biological factors external to a person, and all the related factors impacting behaviours. It encompasses the assessment and control of those environmental factors can potentially affect health. It targeted towards preventing disease and creating health-supportive environments” (13, 14). Children are especially more at risk because they have no control over their prenatal and postnatal environment, including the air, water, food, and their place of residence (9). These threats form the foundation of poor health and often cause and exacerbate other diseases, which may even result in mortality (15). Also, MDG-7, SDG-6, and SDG-7 instruct to enhance the access to improved water sources, improved sanitation, and the use of non-solid fuels to achieve environmental sustainability. A study indicated that there is an enormous variation in water supply and sanitation in different cities of Sub-Saharan Africa (16). Safe water and sanitation practices are significantly related to the child health and mortality in these regions (17). Use of solid fuels for cooking is very high, which was associated with poor health among children (18–20). Various literatures also discussed and empirically shown the weak progress in Sub-Saharan countries in meeting these development goals (21–23). It is also critical to point out that African countries also suffered from various disease outbreaks, such as AIDS and Ebola, which may lead to trim down development efforts (24, 25). As literature suggests, progress toward improving child health and household environmental indicators is better in South Asian regions compared to Sub-Saharan African regions. Child health remains a major public health concern in both the regions. ARI and diarrhea are the major killers of children in South Asia, combined with the high prevalence of underweight. Globally, both the regions constitute a major percentage of child population as well as total number of child deaths. The role of the household environment in influencing child health is important for priority setting the future development goals and agenda in these two regions.

This study explored the key question about the association between household environmental conditions with child survival and health in Sub-Saharan African and South Asian regions in the current scenario. We estimated the risk of death, morbidity, and undernutrition among children living in the households with improved sources of water, sanitation, and non-solid cooking fuel.

## Data Sources

Two sources of information were explored in this study. First, data from World Health Statistics (WHS)-2014 for most of the Sub-Saharan African and South Asian countries were used (26). The WHS has compiled and published data on various developmental indicators in different countries. We used the information on infant mortality, child mortality, population access to the improved sources of water, population access to the improved sources of sanitation, and population using non-solid cooking fuel for South Asian and Sub-Saharan African countries. All the information in WHS-2014 refers to the year 2012.

Demographic and Health surveys (DHSs) are the household surveys that collect and disseminate accurate, nationally representative data on maternal and child health. For assessing the relationship between household environment and child survival, available standard DHS conducted in Sub-Saharan Africa and South Asia countries after 2010 was included in the study, as the second source of information (27). MDGs were signed in the year 2000, and new SDGs were announced in the year 2015. To reflect the current scenario with regard to development goals, we had used DHS done after the year 2010. Non-standard DHSs were excluded either because they had not focused on child health and household environment or may not have been representative of the respective countries. This resulted in the inclusion of 15 countries named Bangladesh (2011), Congo Republic (2013–2014), Cote d’Ivoire (2011–2012), Ethiopia (2011), Mozambique (2011), Mali (2012–2013), Niger (2012), Nigeria (2013), Nepal (2011), Pakistan (2012–2013), Sierra Leone (2013), and Uganda (2011). Sample sizes (total number of children under 5 years old) of the respective countries are given in Table 1.

**Table 1:** **Selected countries and their respective sample sizes**.

Countries	Number of children (*N*)
Bangladesh (2011)	8753
Congo Republic (2013–2014)	18,716
Cote d’Ivoire (2011–2012)	7776
Ethiopia (2011)	11,654
Gambia (2013)	8088
Mali (2012–2013)	10,326
Mozambique (2011)	11,012
Namibia (2013)	5046
Nepal (2011)	5306
Niger (2012)	12,558
Nigeria (2013)	31,482
Pakistan (2012–2013)	11,763
Sierra Leone (2013)	11,938
Uganda (2011)	7878
Zambia (2013)	13,457

## Household Environment Conditions

In DHSs, the information on household environment condition is collected through an exhaustive list. Water and sanitation conditions are classified into two categories as improved and non-improved sources, as per WHO guidelines (28). Use of cooking fuel is classified into two categories as non-solid fuel and solid fuel (18).

## Statistical Analysis

First, the information from the WHS was used to plot the scatter plot diagram. The scatter diagram was plotted for infant and child mortality in the *y*-axis and household environmental indicators on the *x*-axis. The exponential curve was fitted to assess the relationship between access to improved source of water, sanitation, and use of non-solid cooking fuel with the infant and under-five mortality in all of the countries in Sub-Saharan Africa and South Asia.

Demographic and Health Surveys were used for further investigation. Cross tabulation was performed to study the percentage of children living in safe household environment conditions in selected countries. Missing values were excluded from the analysis. Cox regression was used to analyze the survival pattern of children and the effect of the household environment in influencing the survival of children. Cox regression (or proportional hazards regression) is a method for investigating the effect of several variables on the time a specified event takes to happen. In the context of an outcome such as death, this is known as Cox regression for survival analysis. An assumption of the proportional hazard model is that the hazard function for an individual (observation in the analysis) depends on the values of the covariates and the value of the baseline hazard (29). Given two individuals with particular values for the covariates, the ratio of the estimated hazards over time will be constant. Logistic regression was done to calculate the risk of morbidity (diarrhea, fever, and cough) and undernutrition (stunting, wasting, and underweight) by household environment conditions. Household environment risks adjusted for sex of the child, birth order, multiple births, size at birth, mother’s education status, mother’s age at birth, place of residence, and wealth index. The Sub-Saharan countries of Cote d’Ivoire, Congo Republic, Ethiopia, Gambia, Mali, Mozambique, Niger, Sierra Leone, Uganda, and Zambia do not have enough number of children living in the households using non-solid fuel. So, we were not able to calculate the risk in these countries. The likelihood of mortality and morbidity is also known to differ in the first year of life. The analysis was performed separately for children aged 0–12 and 12–59 months. STATA 12 was used to perform all the analysis.

## Results

**Figure 1:**
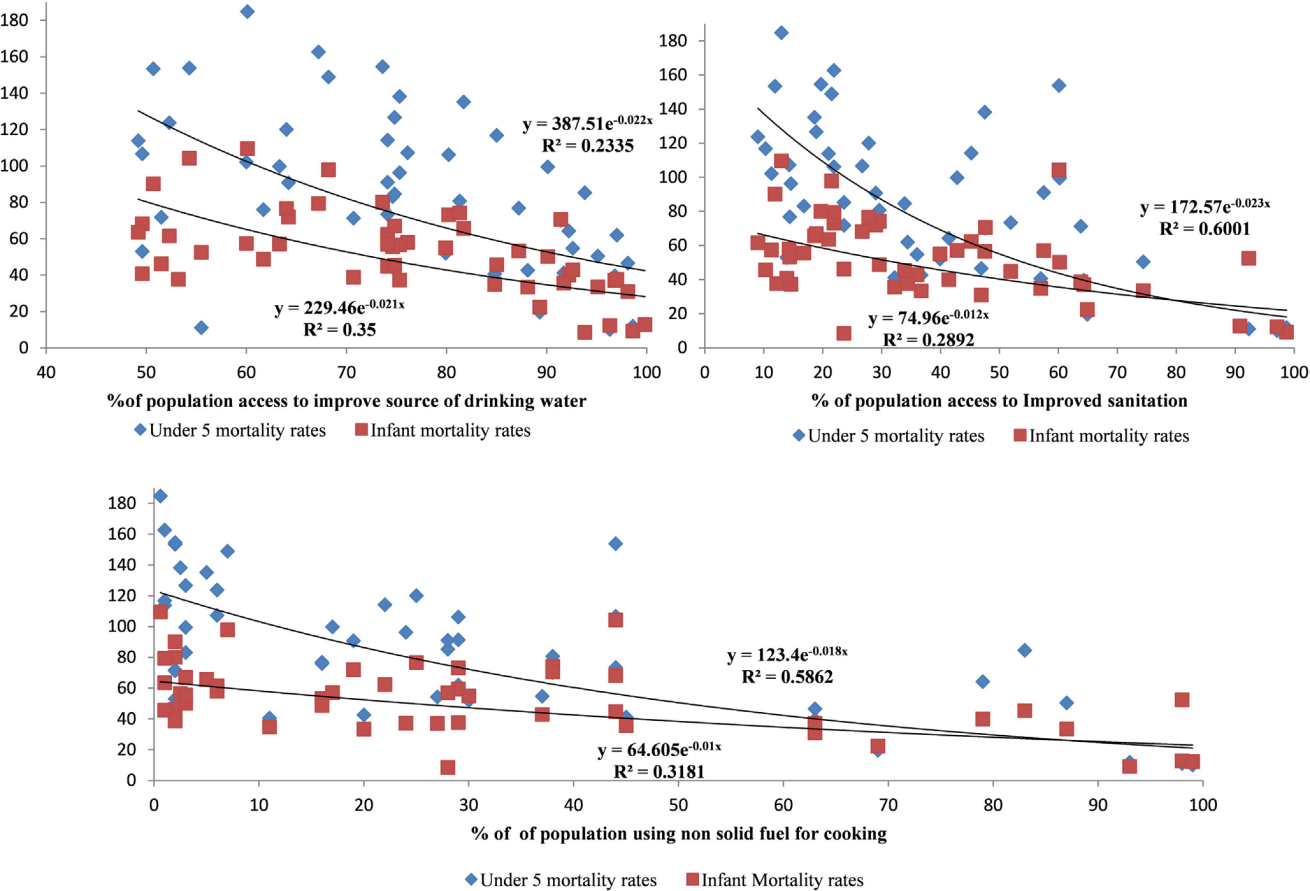
**Relationship between infant and child mortality with access to improved source of water, improved sanitation, and use of non-solid cooking fuel in Sub-Saharan Africa and South Asia**.

Infant and child mortality was related to water and sanitation conditions. As the percentage of persons with improved access to the sources of water and sanitation increased, the infant and child mortality declined (Figure 1). The increasing use of non-solid fuel for cooking was associated with the improved child and infant mortality rates. The exponential curve fitted well with child mortality and environmental indicators, which meant that, as soon as access to water, sanitation, and non-solid cooking fuel improved, there was a rapid reduction in infant and child mortality. Once a high percentage of the population had access to improved water and sanitation, thereafter, it did not influence on child mortality much. All countries in Sub-Saharan Africa or South Asia had more than 45% of the population with access to improved water sources. The percentage of the population using non-solid cooking fuel was in the range of 0–30%. However, the percentage of population access to improved sources of sanitation was well spread out for all of the countries.

**Figure 2:**
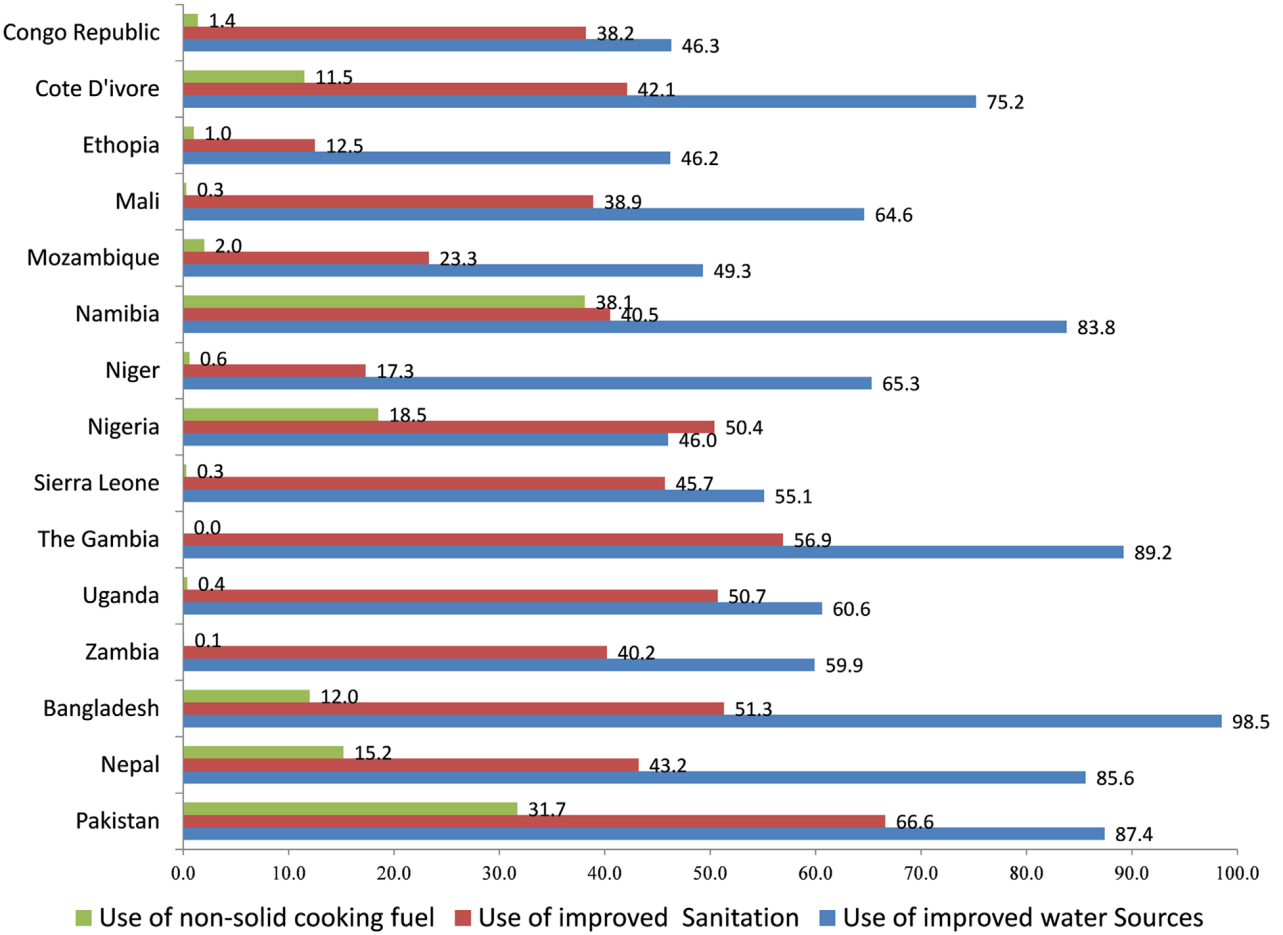
**Percentage of children living in safe household environment (water, sanitation, and cooking fuel) in Sub-Saharan African and South Asian countries**.

Figure 2 shows the percentage of children living in different household environment conditions in selected countries using DHSs. It is not very surprising to find that the percentage of children living in a safe household environment is high in South Asian countries, as compared to Sub-Saharan Africa, as South Asian countries are economically more developed than Sub-Saharan Africa. In most countries studied, there were a high percentage of children living in households with improved water sources. The percentage of children living in the household using non-solid cooking fuel was very low in all the countries. It was almost negligible in countries like Ethiopia, Mali, Niger, Sierra Leone, and Uganda.

The Tables 2 and 3 showed the risk of death and morbidity in household environmental conditions. None of the household environmental conditions were significantly related to child survival at age less than 12 months (Table 2). In the age group 0–12 months, the use of improved sanitation was significantly related to the lower odds ratio of diarrhea in Pakistan (OR = 0.754) and lower odds ratio of fever in the Congo Republic (OR = 0.774) and Uganda (OR = 0.655) compared to the households without access to improved sanitation. Use of improved water sources had significantly lower risk of diarrhea in Nigeria (OR = 0.836) and Sierra Leone (OR = 0.732) compared to the households without access to improved water sources. Assessment of the use of non-solid cooking fuel was done only in few countries but significantly related to cough in Nigeria (OR = 0.648) among children aged 0–12 months. In the age group of 12–59 months (Table 3), the use of improved sanitation was significantly related to increasing child survival in the Congo Republic (OR = 0.779) and Niger (OR = 0.631). Use of improved source of water also resulted in significantly reduced risk of death among children aged 12–59 months in the Congo Republic (OR = 0.712) and Gambia (OR = 0.467). Use of improved source of water had significantly lower risk of fever in the Congo Republic (OR = 0.791) and lower risk of diarrhea in Sierra Leone (OR = 0.827). Use of improved sanitation was related to lower risk of fever in the Congo Republic (OR = 0.818), Mali (0.762), and Uganda (OR = 0.889), when compared to no improvement in sanitation. Use of non-solid cooking fuel was found to significantly reduce the risk of fever in Namibia (OR = 0.742) and Bangladesh (OR = 0.778) compared to the use of solid cooking fuels.

**Table 2:** **Household environment conditions in children aged less than 12 months: adjusted risk of death and morbidity outcomes in selected South Asian and Sub-Saharan African countries**.

Countries	Household environment conditions	Risk of death	Odds ratio of diarrhea	Odds ratio of fever	Odds ratio of cough
Cote d’Ivoire	Improved sources of water	1.078	1.048	0.876	0.919
	Improved sources of sanitation	1.02	0.952	0.893	0.888
Congo Republic	Improved sources of water	0.891	0.986	0.881	1.042
	Improved sources of sanitation	0.86	0.877	0.774***	0.856*
Ethiopia	Improved sources of water	0.89	0.899	0.91	0.904
	Improved sources of sanitation	0.822	0.869	0.97	0.869
Gambia	Improved sources of water	0.74	0.731*	0.884	0.952
	Improved sources of sanitation	1.011	1.513**	1.166	1.409*
Mali	Improved sources of water	1.241	1.374	1.027	0.765
	Improved sources of sanitation	0.737	0.978	1.047	0.982
Mozambique	Improved sources of water	1.193	1.101	1.125	1.384*
	Improved sources of sanitation	1.175	1.045	0.918	0.881
Namibia	Improved sources of water	1.469	0.969	1.021	0.835
	Improved sources of sanitation	0.724	0.739	1.163	0.864
	Use of non-solid cooking fuel	0.683	0.978	0.884	0.879
Nigeria	Improved sources of water	0.945	0.836**	0.934	0.771***
	Improved sources of sanitation	0.947	1.290**	1.275*	0.943
	Use of non-solid cooking fuel	0.816	0.648**	0.551***	0.921
Niger	Improved sources of water	0.794*	0.826	0.826	0.791*
	Improved sources of sanitation	0.733	0.829	0.907	0.872
Sierra Leone	Improved sources of water	1.106	0.732**	0.847	0.868
	Improved sources of sanitation	0.901	0.891	0.911	0.858
Uganda	Improved sources of water	1.158	1.022	1.476**	0.824
	Improved sources of sanitation	0.775	0.956	0.655**	0.977
Zambia	Improved sources of water	1.18	1.093	0.896	1.028
	Improved sources of sanitation	1.178	0.951	1.092	1.107
Bangladesh	Improved sources of water	0.982	0.87	0.631	0.555*
	Improved sources of sanitation	1.200	1.114	1.024	1.052
	Use of non-solid cooking fuel	0.727	1.376	0.850	0.827
Nepal	Improved sources of water	1.418	1.129	1.282	1.187
	Improved sources of sanitation	1.133	0.994	1.162	1.185
	Use of non-solid cooking fuel	0.962	0.899	1.321	2.182**
Pakistan	Improved sources of water	0.965	1.23	1.163	1.119
	Improved sources of sanitation	0.813	0.754**	0.998	1.085
	Use of non-solid cooking fuel	1.460	0.128	1.22	1.273

*Adjusted for sex of the child, birth order, multiple births, size at birth, mother’s education status, mother’s age at birth, place of residents, and wealth index*.

*p < 0.10.

**p < 0.05.

***p < 0.01.

**Table 3:** **Household environment conditions in children aged 12–59 months: adjusted risk of death and morbidity outcomes in selected South Asian and Sub-Saharan African countries**.

Countries	Household environment conditions	Risk of death	Odds ratio of diarrhea	Odds ratio of fever	Odds ratio of cough
Cote d’Ivoire	Improved sources of water	0.937	1.03	0.802***	0.887
	Improved sources of sanitation	0.888	0.855*	1.0674	0.846*
Congo Republic	Improved sources of water	0.712***	0.929	0.898**	0.942
	Improved sources of sanitation	0.779**	0.791***	0.818***	0.894***
Ethiopia	Improved sources of water	0.843	0.985	1.035	0.968
	Improved sources of sanitation	1.153	0.905	0.979	0.874
Gambia	Improved sources of water	0.467**	1.066	1.088	0.978
	Improved sources of sanitation	1.277	1.132	1.021	1.343**
Mali	Improved sources of water	1.089	1.272**	1.333**	1.117
	Improved sources of sanitation	0.942	0.871	0.762**	0.887
Mozambique	Improved sources of water	1.106	1.029	0.929	1.19
	Improved sources of sanitation	1.104	1.029	0.914	1.184
Namibia	Improved sources of water	2.422	0.996	1.299	1.219*
	Improved sources of sanitation	0.447	1.111	0.899	0.918
	Use of non-solid cooking fuel	0.898	0.549***	0.742***	0.865*
Nigeria	Improved sources of water	1.081	0.938	0.933	0.874***
	Improved sources of sanitation	0.091	1.148**	1.109**	0.998
	Use of non-solid cooking fuel	0.787	0.759**	0.767*	1.03
Niger	Improved sources of water	0.959	1.036	0.865*	0.922
	Improved sources of sanitation	0.631**	0.887	1.151	1.028
Sierra Leone	Improved sources of water	0.951	0.827**	1.034	1.059
	Improved sources of sanitation	1.11	1.104	0.998	0.942
Uganda	Improved sources of water	0.838	1.084	1.313***	1.135*
	Improved sources of sanitation	1.155	0.919	0.889**	1.001
Zambia	Improved sources of water	0.953	1.042	1.016	1.072
	Improved sources of sanitation	1.323	0.994	0.97	0.926
Bangladesh	Improved sources of water	–	0.915	0.824	0.657**
	Improved sources of sanitation	0.637	0.953	1.063	1.044
	Use of non-solid cooking fuel	1.679	1.305	0.778**	0.924
Nepal	Improved sources of water	1.068	1.079	1.107	1.087
	Improved sources of sanitation	2.606*	0.859	1.103	0.977
	Use of non-solid cooking fuel	1.452	0.936	0.731	1.141
Pakistan	Improved sources of water	1.714	0.961	0.955	0.943
	Improved sources of sanitation	0.851	0.923	1.023	1.104
	Use of non-solid cooking fuel	0.742	1.003	1.025	1.067

*Adjusted for sex of the child, birth order, multiple births, size at birth, mother’s education status, mother’s age at birth, place of residents, and wealth index*.

*p < 0.10.

**p < 0.05.

***p < 0.01.

We further investigated the role of these household environmental conditions with nutritional indicators, such as stunting, wasting, and underweight, among children. There was a poor association with nutrition among children aged 0–12 months (Table 4). The household environmental conditions were found significantly influencing nutrition among children aged 12–59 months in most of the countries. (Table 5). Use of improved sources of water was significantly related to the lower odds ratio of stunting (0.767) and wasting in Uganda (0.500), while it was found significantly reducing odds ratio of underweight in Bangladesh (OR = 0.594) and Mali (OR = 0.768) compared to the use of non-improved water sources. Use of improved sanitation significantly related to lower risk of underweight in the Congo Republic (OR = 0.821), Ethiopia (OR = 0.811), and Mali (OR = 0.768). It was also associated with lower risk of wasting in Nepal (OR = 0.634) and lower risk of stunting in Cote d’Ivoire (OR = 0.709) and Ethiopia (OR = 0.854). Use of non-solid fuel was also significantly related to lower risk of stunting (OR = 0.749) and underweight (OR = 0.849) in Namibia.

**Table 4:** **Undernutrition among children aged less than 12 months: adjusted odds ratio by household environment conditions in selected South Asian and Sub-Saharan African countries**.

Countries	Household environment conditions	Odds ratio of stunting	Odds ratio of wasting	Odds ratio of underweight
Cote d’Ivoire	Improved sources of water	0.505***	0.861	0.781
	Improved sources of sanitation	0.932	0.909	1.004
Congo Republic	Improved sources of water	1.043	1.2	0.803
	Improved sources of sanitation	1.064	0.964	0.786
Ethiopia	Improved sources of water	0.957	1.155	1.207
	Improved sources of sanitation	0.446**	0.984	0.846
Gambia	Improved sources of water	1.05	0.999	1.096
	Improved sources of sanitation	1.001	0.583**	0.764
Mali	Improved sources of water	0.824	1.381	0.793
	Improved sources of sanitation	1.234	1.282	1.164
Mozambique	Improved sources of water	1.029	0.877	1.169
	Improved sources of sanitation	1.16	0.733	1.843
Namibia	Improved sources of water	–	0.644	–
	Improved sources of sanitation	–	0.667	–
	Use of non-solid cooking fuel	–	0.768	–
Nigeria	Improved sources of water	1.074	0.951	1.006
	Improved sources of sanitation	1.228**	1.292***	1.484***
	Use of non-solid cooking fuel	0.972	1.032	0.951
Niger	Improved sources of water	1.042	1.071	1.099
	Improved sources of sanitation	1.305	0.811	0.874
Sierra Leone	Improved sources of water	0.857	1.269	0.887
	Improved sources of sanitation	1.189	0.724	0.86
Uganda	Improved sources of water	1.052	1.862	1.283
	Improved sources of sanitation	1.016	0.881	0.873
Zambia	Improved sources of water	0.917	1.542**	0.976
	Improved sources of sanitation	1.072	1.089	0.957
Bangladesh	Improved sources of water	1.076	1.001	1.048
	Improved sources of sanitation	1.131	0.948	0.902
	Use of non-solid cooking fuel	1.225	1.248	1.027
Nepal	Improved sources of water	1.657	0.599	0.776
	Improved sources of sanitation	0.596	0.9	0.706
	Use of non-solid cooking fuel	1.168	1.088	0.608
Pakistan	Improved sources of water	0.742	1.532	1.318
	Improved sources of sanitation	1.365	0.737	0.991
	Use of non-solid cooking fuel	1.755	0.742	1.968*

*Adjusted for sex of the child, birth order, multiple births, size at birth, mother’s education status, mother’s age at birth, place of residents, and wealth index*.

*p < 0.10.

**p < 0.05.

***p < 0.01.

**Table 5:** **Undernutrition among children aged 12–59 months: adjusted odds ratio by household environment conditions in selected South Asian and Sub-Saharan African countries**.

Countries	Household environment conditions	Odds ratio of stunting	Odds ratio of wasting	Odds ratio of underweight
Cote d’Ivoire	Improved sources of water	1.181	0.99	1.492*
	Improved sources of sanitation	0.709***	0.713	0.751*
Congo Republic	Improved sources of water	1.073	1.064	0.938
	Improved sources of sanitation	0.870**	1.109	0.811***
Ethiopia	Improved sources of water	0.956	1.158*	1.06
	Improved sources of sanitation	0.854**	0.847	0.821**
Gambia	Improved sources of water	1.218	0.653**	0.962
	Improved sources of sanitation	0.882	1.201	0.996
Mali	Improved sources of water	0.911	0.699***	0.768***
	Improved sources of sanitation	0.997	0.692**	0.964
Mozambique	Improved sources of water	0.896*	1.394*	0.993
	Improved sources of sanitation	0.888*	0.979	0.98
Namibia	Improved sources of water	0.963	1.025	0.999
	Improved sources of sanitation	0.786	0.809	1.133
	Use of non-solid cooking fuel	0.749***	0.942	0.849**
Nigeria	Improved sources of water	1.015	1.193**	1.151**
	Improved sources of sanitation	1.142***	1.491**	1.321**
	Use of non-solid cooking fuel	0.651*	1.11	0.636
Niger	Improved sources of water	1.191*	1.005	1.11
	Improved sources of sanitation	0.928	0.934	0.96
Sierra Leone	Improved sources of water	0.974	0.876	0.805*
	Improved sources of sanitation	1.078	0.793	0.728***
Uganda	Improved sources of water	0.767**	0.500**	0.769
	Improved sources of sanitation	1.113	0.623	1.003
Zambia	Improved sources of water	0.924	0.904	0.969
	Improved sources of sanitation	1.199**	0.872	1.003
Bangladesh	Improved sources of water	0.839	0.640**	0.594***
	Improved sources of sanitation	0.889*	1.023	0.901*
	Use of non-solid cooking fuel	1.103	1.255	1.089
Nepal	Improved sources of water	0.87	0.914	0.777*
	Improved sources of sanitation	1.347**	0.634**	0.861
	Use of non-solid cooking fuel	0.896	1.263	0.771
Pakistan	Improved sources of water	1.177	0.801	1.278*
	Improved sources of sanitation	1.141	0.855	0.859
	Use of non-solid cooking fuel	0.918	1.213	1.398**

*Adjusted for sex of the child, birth order, multiple births, size at birth, mother’s education status, mother’s age at birth, place of residents, and wealth index*.

*p < 0.10.

**p < 0.05.

***p < 0.01.

## Discussion

### Development Goals and the Association between Household Environment and Child Health

Millennium Development Goals brought the importance of health system strengthening, improving social, economic, and environmental conditions, which determined poor health conditions in developing countries. Studies indicate that there is a remarkable improvement in infant and child mortality, especially in South Asia (30). All regions, except Sub-Saharan Africa, have successfully halved the under-five mortality rate compared to 1990 in recent years (30, 31). Access to safe drinking water (one of the goals of MDG-7) is also well on track except for Sub-Saharan African region (31, 32). Water, sanitation, and the use of cooking fuel are known to influence child morbidity and nutrition, which, in turn, increases child mortality in developing countries (11–13, 16, 33). Our study shows that water and sanitation condition had the significant impact on child survival in some of the African countries. However, studies do suggest that many Sub-Saharan countries will not be able to achieve these development goals (23, 32). Sub-Saharan African countries were also severely affected by epidemics, such as HIV/AIDS and Ebola, in recent years, which caused high mortality in the region.

The access to non-solid fuel was very low in some of the African countries, and the relationship cannot be assessed. Using clean energy also has an economic perspective. The widespread poverty and economic inequality could be the reason for the high use of solid fuel in these countries (34). However, in South Asian countries, the relationship between household environment and child survival were not found significant. The reason could be that the South Asian countries have achieved greater access to safer water and sanitation conditions (more considerable progress toward MDG-7). Although, the situation in India was not assessed due to the lack of a recent DHS, which may shows a likely influence in South Asia. The literature and different studies in India had shown the reduction in child mortality as well as an improvement in access to improved water and sanitation facilities (35, 36). Bangladesh has also shown great progress in child health and household environmental indicators in recent decades (37, 38). The recent earthquakes in Nepal will influence the availability and accessibility of water and sanitation. Development efforts in Nepal need to rebuild health facilities and household environment conditions, which were destroyed by the earthquake.

### Health System and Development Goals

Achievements of these goals are also highly affected by the governance of health system in the respective countries (39). Travis et al. explains the different interventions, the health system responses, and the constraints of these responses in addressing these goals (40). Some of these constraints include financial–physical inaccessibility, inappropriate skills or poorly motivated health staff, and poor quality of health service deliveries (39, 40). Child survival strategies in developing countries focus on case management and treatment. These strategies have focused on resisting or reducing infection once it occurred rather than reducing the exposure to these environmental threats (40, 41). The importance of improving environmental surroundings holds a greater importance in enhancing health conditions in these regions (41). As our finding also suggest that, grown-up children (aged 12–59 months) will be more exposed to these environmental threats and also more at risk of mortality, morbidity, and undernutrition due to these conditions. The study has provided evidence that these environmental conditions still hold important for improving child health. However, more in-depth quantitative or qualitative analysis of each country will bring out appropriate measures of intensifying development efforts in the respective countries.

There are some limitations of this study. Indicators and data for particular countries may differ by data quality, which can affect results and comparisons. DHSs are cross-sectional household surveys, so the direction of association cannot be established. India was not part of the study, which may lead to under-representation of South Asia.

## Conclusion

Surveys indicate that a high proportion of children are living in poor household environmental conditions in the South Asian as well as in Sub-Saharan African countries. There is a positive correlation existing between improving access to safe drinking water and sanitation with under-five and infant mortality. It is acknowledged that these can be a result of overall efforts of development and improving health conditions in these countries. The use of non-solid cooking fuel is very high in most of the Sub-Saharan African and South Asian countries. Poor environmental conditions, such as water and sanitation conditions, had a significant influence on child survival in some of the Sub-Saharan African countries but did not have much influence in the case of South Asian countries.

## Author Contributions

AA performed the data analysis and writing of the manuscript. AA and NR participated in improving and finalizing manuscript.

## Conflict of Interest Statement

The authors declare that the research was conducted in the absence of any commercial or financial relationships that could be construed as a potential conflict of interest.
